# Double Photocrosslinked Responsive Hydrogels Based on Hydroxypropyl Guar

**DOI:** 10.3390/ijms242417477

**Published:** 2023-12-14

**Authors:** Alexander S. Ospennikov, Andrey V. Shibaev, Olga E. Philippova

**Affiliations:** Physics Department, Moscow State University, Moscow 119991, Russia; ospennikov@polly.phys.msu.ru (A.S.O.); shibaev@polly.phys.msu.ru (A.V.S.)

**Keywords:** hydrogel, crosslinking, rheology, polymer network, hydroxypropyl guar

## Abstract

Double crosslinked hydrogels based on a biodegradable polymer were prepared by photocopolymerization of methacrylated hydroxypropyl guar (HPG-MA) and 3-acrylamidophenylboronic acid. Along with irreversible strong covalent crosslinks by methacrylic groups, these hydrogels contained labile boronate crosslinks formed as a result of the interaction of boronic acid with cis-diol moieties of HPG. These hydrogels demonstrated higher elasticity than HPG-MA hydrogels with only irreversible covalent crosslinks. Labile boronate crosslinks not only strengthened the hydrogels but also imparted to them pronounced responsive properties. It was demonstrated that the mechanical properties, the swelling behavior, as well as the uptake and release of some substances from the double crosslinked hydrogel were pH controlled. For instance, the hydrogels could release cationic disinfectant at a rate regulated by pH. Such photocrosslinkable in situ forming hydrogels are very promising for the production of smart coatings that release targeted substances at the desired rate.

## 1. Introduction

Hydrogels consist of crosslinked polymers swollen in water [1,2]. Due to their high water content and porous structure responsive to a number of physiological stimuli such as pH or temperature, these soft materials are widely used in wound dressings, tissue engineering, biosensing, delivery of drugs and antiseptics, etc. [3,4,5,6]. In particular, several anionic synthetic hydrogels (copolymers of acrylamide and sodium 2-acrylamido-2-methylpropane sulfonate, copolymers of acrylamide and sodium methacrylate, and copolymers of vinylpyrrolidone and sodium methacrylate) were proposed [7] as coatings for long-lasting release of cationic disinfectants effective against SARS-CoV-2 [8]. However, crosslinked polymers are insoluble and infusible, which hampers their removal from substrates [9]. In the last few years, much attention has been paid to the recovery or recycling of crosslinked polymers because of environmental concerns [9].

From an ecological point of view, of particular interest for gel preparation are naturally occurring polymers that can offer such properties as biocompatibility, biodegradability, and non-toxicity [10]. Among naturally occurring polymers, hydrogels of various polysaccharides, including agarose, alginate, and κ-carrageenan, have been used for many biomedical applications [11]. Some other polysaccharides, like guar gum and its derivatives, are less applied for this purpose, but they have great potential because of their wide availability and low price [12,13].

Guar gum is derived from the seeds of the guar plant (*Cyanaposis tetragonolobus* L.), which grows mainly in India and Pakistan. Guar is an uncharged linear galactomannan composed of a (1→4)-β-D-mannopyranose backbone with (1→6)-α-D-galactopyranosyl residues attached as side groups [12]. The molar mass of guar is one of the highest among all naturally occurring water-soluble polysaccharides [12,14]; therefore, rather low polymer concentrations are needed to form semi-dilute entangled solutions, which are required for gel formation.

**Figure 1:**
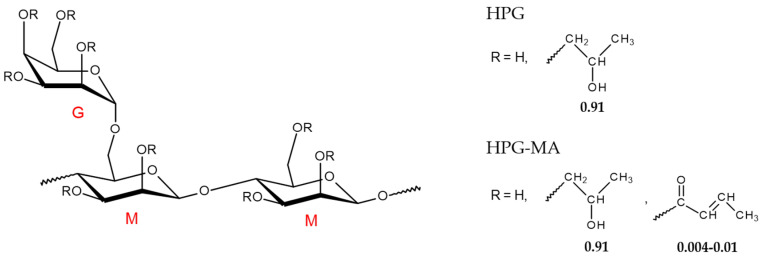
Molecular structure of hydroxypropyl guar (HPG) and methacrylated hydroxypropyl guar (HPG-MA). The degree of substitution by hydroxypropyl and methacrylate groups is indicated in the figure.

To improve the hydration of guar at ambient temperatures, it can be chemically modified [15]. The most widely available derivative of natural guar is hydroxypropyl guar (HPG) [16] (Figure 1). It has better water solubility because the hydroxypropyl groups hinder hydrogen bonding between guar macromolecules, thereby reducing intermolecular aggregation [16]. At the same time, it remains a biocompatible and biodegradable polymer [17].

To obtain hydrogels, HPG is most often crosslinked by borate ions, which form dynamic covalent bonds with the 1,2- and 1,3-cis-diol groups of HPG [18,19,20,21]. A detailed mechanism of the borate–diol interaction is described in ref. [22]. The free energy of the reaction between guar and the borate anion was estimated as −2.7 kcal/mol [23], which is only a few kT. Therefore, such crosslinks are very labile (reversible) even at room temperature and impart to the gel very pronounced responsive properties with respect to many external stimuli (pH, temperature, ionic strength, and various additives like glucose and so forth). Such hydrogels are prospective matrices for regulating the release of drugs or antiseptics in response to specific triggers, which can reduce potential toxic effects. At the same time, gels with labile crosslinks possess rather poor mechanical properties, which limits their applications. To obtain mechanically robust gels, irreversible chemical crosslinking should be used.

An effective strategy for producing irreversible polymer crosslinking is photopolymerization [24]. It provides good spatial and temporal control over crosslinking, is non-invasive, and allows the possibility of in situ polymerization [25]. It is used to produce strong coatings on various surfaces, even very sophisticated ones [26,27]. Also, it is widely employed in several techniques of 3D printing to fabricate objects according to computer-aided design based on digital model files [28,29,30]. Moreover, it is attracting growing interest in the development of in situ gelling hydrogels for drug delivery, biosensing, and tissue engineering [31,32]. In this case, aqueous solutions of monomers or macromonomers act as injectable materials, remaining in liquid state before applying a light source and becoming hydrogels after exposure to UV-visible light of a specific wavelength [33].

For photocrosslinking, functional groups (e.g., vinyl groups) participating in polymerization should be incorporated into polysaccharide chains [32]. For this aim, for instance, methacrylated guar macromonomers have been synthesized [34]. Sometimes methacrylated derivatives are used to produce networks based on guar grafted with synthetic polymer chains, e.g., poly(sodium acrylate) [35] or acrylamide [36]. Hydrogels crosslinked by irreversible chemical bonds are stronger than those crosslinked with labile bonds; however, they are much less responsive to external triggers.

In this paper, we propose to combine two types of crosslinks in the same gel in order to obtain a strong hydrogel that is yet highly responsive to the environment. Both types of crosslinks will be incorporated into the HPG network during photopolymerization. Strong irreversible crosslinks will be formed by photopolymerization of methacrylate groups attached to HPG via chemical modification using methacrylic anhydride (Figure 1). To provide labile crosslinks, HPG will be grafted with poly(3-(acrylamido)phenylboronic acid) (PAAPBA) chains, which will be formed from 3-(acrylamido)phenylboronic acid (AAPBA) monomer concurrently with covalent crosslinking during photopolymerization. The phenylboronic acid (PBA) groups of the PAAPBA chains will be able to link to the 1,2- and 1,3-cis-diol moieties of HPG, forming reversible boronate crosslinks [37]. By comparing against hydrogels containing only irreversible crosslinks, it is demonstrated that the combination of two types of crosslinks, on the one hand, strengthens the gel, and on the other hand, provides responsiveness to pH. On the basis of the double crosslinked gel, a pH-sensitive polymer coating is prepared by one-pot photopolymerization. It is shown that the gel can deliver a cationic antiseptic agent, cetylpyridinium chloride (CPC) [7,8], at a pH-controlled rate.

## 2. Results and Discussion

### 2.1. Synthesis of Photopolymerizable Polysaccharide Macromonomer

**Figure 2:**
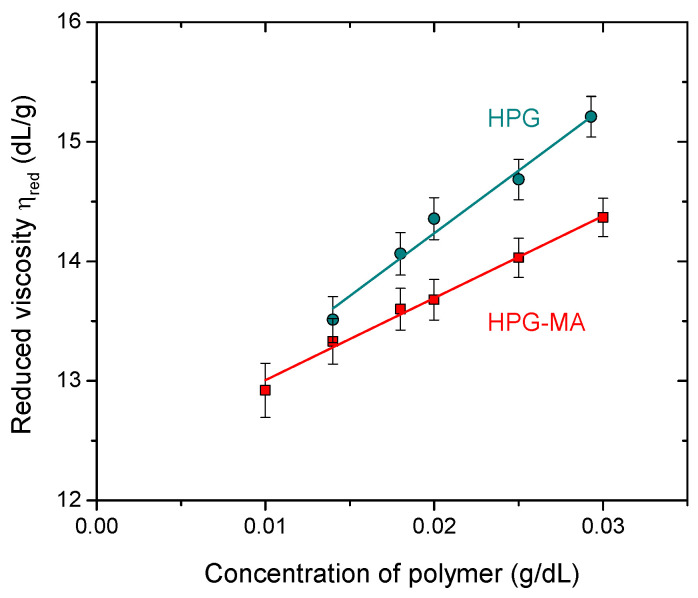
Reduced viscosity versus polymer concentration for aqueous solutions of hydroxypropyl guar (HPG, circles) and methacrylated hydroxypropyl guar (HPG-MA, squares) at 20 °C.

The initial compound for the synthesis of the hydrogel under study was HPG, which has an average of 0.91 hydroxypropyl groups per monosaccharide residue and a galactose to mannose ratio of 0.7, as was previously determined by ^1^H NMR [37]. The molar mass of the HPG under study was equal to 1,500,000 g/mol. This value was estimated from the intrinsic viscosity [η] of HPG solution in water ([η] = 12.14 dL/g, Figure 2), using the Mark–Houwink–Sakurada equation [η] = 1.72 × 10^−4^ M_w_^0.785^ (dL/g) with the coefficients determined by Cheng et al. [16]. The obtained value of the molar mass corresponded with the degree of polymerization of ca. 4100 (per one mannopyranose ring in the backbone).

**Figure 3:**
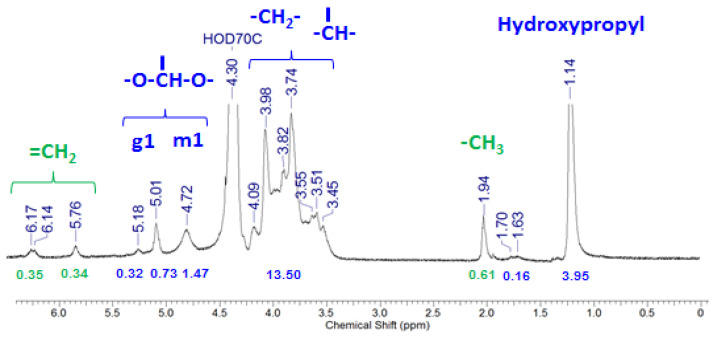
^1^H NMR spectrum of 1 wt% solution of HPG-MA (degree of modification 0.08) in D_2_O. Peak assignment according to ref. [36] is given on the figure.

To crosslink the HPG chains with one another, they were chemically modified with the introduction of photopolymerizable methacrylate groups to yield HPG-MA. Methacrylation was performed by the reaction of HPG with methacrylic anhydride at 4 °C. The incorporation of methacrylic groups into the polymer chains was confirmed by ^1^H NMR data for samples thoroughly washed by dialysis to remove unreacted methacrylic acid and methacrylic anhydride. A typical spectrum is presented in Figure 3. It contains methacrylate peaks at δ 6.14–6.17, 5.76, and 1.94 ppm [38], marked by green. The degree of modification (DM, the average number of methacrylate substituents per one monosaccharide unit) was determined from the ratio of the integral of the peak of the methacrylate CH_3_ group protons (at δ 1.94 ppm) corresponding to one proton to the sum of the integrals of the peaks of the monosaccharide anomeric protons –O-CH-O- of the galactopyranosyl (at δ 5.18 and 5.01 ppm [39]) and mannopyranosyl rings (at δ 4.72 ppm [39]). Since each methacrylate group contains three CH_3_ group protons and each monosaccharide unit contains one anomeric proton, one can estimate the degree of modification (DM) as follows:

DM=ImethacrylateImonosaccharide=13I1.94 ppmI5.18 ppm+I5.01 ppm+I4.72 ppm

where $I_{methacrylate}$ is the integral corresponding to one proton of the methyl group in methacrylate moieties, and $I_{monosaccharide}$ is the integral of the anomeric protons of the monosaccharide units.

**Figure 4:**
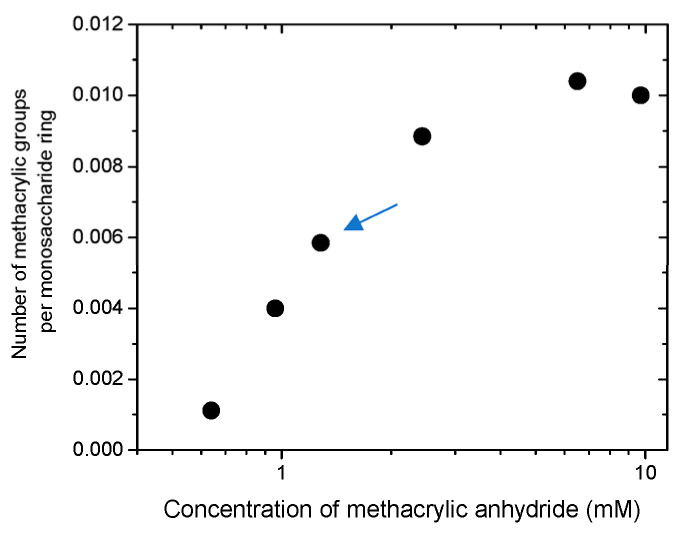
Dependence of the number of methacrylic groups incorporated into HPG-MA (per monosaccharide ring) on the concentration of methacrylic anhydride added to 0.5 wt% solution of HPG at pH 8 during chemical modification. The sample used for further studies is marked with an arrow. It contains 0.56 mol% methacrylic groups (one methacrylic group per 106 mannopyranose rings in the backbone).

Photopolymerizable HPG-MA macromonomers with different degrees of methacrylation were prepared by varying the concentration of methacrylic anhydride in the reaction mixture (Figure 4). Since the methacrylate groups were further used for the crosslinking of polysaccharide chains, a rather small fraction of units was modified in order to provide rather long subchains in the network. For further studies, an HPG-MA sample was prepared containing 0.56 mol% methacrylic groups (one methacrylic group per 180 monosaccharide rings or per 106 mannopyranose rings in the backbone).

The molar mass of the modified polymer HPG-MA was determined by viscometry (Figure 2) from the intrinsic viscosity value ([η] = 12.32 dL/g) using the same coefficients in the Mark–Houwink–Sakurada equation as for unmodified HPG [16]. The obtained value of the molar mass was 1,530,000 g/mol, which was close to that of HPG before modification. Therefore, the methacrylation reaction was not accompanied by the destruction of the polymer.

At the same time, a comparison of the straight lines approximating the dependencies of the reduced viscosity $\left(\eta_{red}\right)$ on the polymer concentration (C) for HPG before and after modification showed that the slope of the line for unmodified HPG solution was much steeper (Figure 2). From the slopes, the values of the dimensionless Huggins coefficient $\left(k_{H}\right)$ were determined using the Huggins relationship [16]:

$$\frac{\eta_{sp}}{C}=\eta_{red}=\left[\eta \right]+k_{H}{\left[\eta \right]}^{2}C$$

They were equal to 0.71 and 0.45 for HPG and HPG-MA solutions, respectively. The Huggins coefficient is known to be a measure of polymer–polymer interactions in dilute solution [39]. Therefore, much lower $k_{H}$ values in the case of HPG-MA indicated weakening of the intermolecular attraction upon polymer modification. This may be due to steric hindrance imposed by bulky methacrylic groups to interpolymer hydrogen bonding, which is one of the main reasons for the association of HPG macromolecules [16]. Note that $k_{H}$ values are around 0.7 at theta conditions and decrease due to the excluded volume effect, reaching a limiting value of ca. 0.3 at good solvent conditions [40]. Therefore, water could be considered as almost a theta solvent for the HPG sample under study and a good solvent for HPG-MA at 20 °C.

### 2.2. HPG-MA Hydrogels

To obtain HPG-MA hydrogels, the methacrylated macromonomer HPG-MA was photocrosslinked by UV irradiation in water in the presence of a biocompatible [41] photo-initiator, lithium phenyl-2,4,6-trimethylbenzoylphosphinate (TPO-Li). The photopolymerization procedure was previously elaborated for several methacrylated polysaccharides: hyaluronic acid [38,41,42], chitosan [41], alginate [42,43], chondroitin sulfate [44], and guar [34]. Here, we used it for the first time for HPG-MA.

For crosslinking, HPG-MA solutions with concentrations ranging from 1 to 8 wt% were used. These concentrations corresponded to an entangled semi-dilute regime, since the entanglement concentration (C_e_) was 0.2 wt%, as was previously determined from rheological data [37]. During UV irradiation, the HPG-MA macromonomers underwent free radical photopolymerization leading to the crosslinking of the polysaccharide chains. As a result of the crosslinking, the viscous liquid HPG-MA was transformed into a hydrogel.

**Figure 5:**
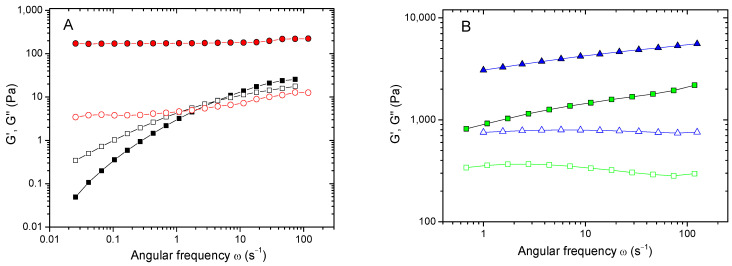
(**A**) Frequency dependencies of storage G′ (filled symbols) and loss G″ (open symbols) moduli for 1 wt% solution of HPG-MA macromonomer with 0.56 mol% MA before (squares) and after (circles) UV irradiation at 20 °C. (**B**) Frequency dependencies of storage G′ (filled symbols) and loss G″ (open symbols) moduli for HPG-MA hydrogels with 0.56 mol% MA at different polymer contents, 5 wt% (squares) and 8 wt% (triangles), at 20 °C.

The gel formation was confirmed by rheological data (Figure 5). Indeed, the initial HPG-MA solution was liquid-like, since the viscous contribution to the complex modulus (the loss modulus G″) exceeded the elastic contribution (the storage modulus G′) at most of the studied frequencies. By contrast, upon irradiation, the storage modulus (G′) became higher than the loss modulus (G″) by almost two orders of magnitude and nearly independent of frequency, demonstrating a wide rubbery plateau (Figure 5).

From the plateau modulus (G_0_), the mesh size of the gels (the size of the unit cell of the network representing the linear distance between two adjacent crosslinks [45]) was estimated by using the formula proposed by MacKintosh et al. [46] for entangled solutions of semiflexible polymers:

$$G_{0}\approx \frac{k_{B}Tl_{p}^{7/5}}{\xi^{22/5}}$$

where $k_{B}$ is the Boltzmann constant, T is the temperature, and $l_{p}$ is the persistence length. For 1 wt% HPG-MA hydrogel, the mesh size ξ was equal to 20.4 nm if $l_{p}$ = 10 nm, according to ref. [47].

Keeping the DM constant, the plateau modulus of HPG-MA hydrogels could be increased from 0.2 up to 5 kPa by increasing the macromonomer concentration (Figure 5). For 8 wt% HPG-MA hydrogel, the plateau modulus was 5 kPa (Figure 5B), which was almost 3-fold higher than that of 8 wt% hyaluronic acid-MA hydrogel [42]. Therefore, the crosslinking in the present system was more effective, which may be due, in particular, to the difference in the structures of hyaluronic acid and HPG. The mesh sizes calculated from G_0_ of HPG-MA hydrogels prepared at different macromonomer concentrations ranged from 10 to 20.4 nm, which is comparable to the porosity of many biological hydrogels [48] and allows for the unrestricted transport of nutrients, metabolism products, small-molecule drugs, antiseptics, and growth factors.

Thus, a series of HPG-MA hydrogels differing in mechanical properties and pore size was prepared by photopolymerization of HPG-MA macromonomer.

### 2.3. HPG-MA-PBA Hydrogels

**Figure 6:**
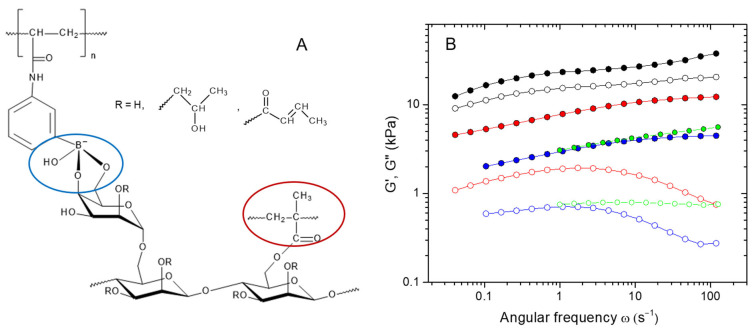
(**A**) Molecular structure of two types of crosslinks in a double crosslinked HPG-MA-PBA network. Crosslinks are marked by ovals. (**B**) Frequency dependencies of storage G′ (filled symbols) and loss G″ (open symbols) moduli for 8 wt% HPG-MA-PBA hydrogels with fixed content of MA (0.56 mol%) and different content of AAPBA: 0% (green symbols), 0.2 wt% (blue symbols), 0.5 wt% (red symbols), and 2 wt% (black symbols).

To synthesize HPG-MA-PBA hydrogels, an aqueous solution containing HPG-MA macromonomer, AAPBA monomer, and a photo-initiator, TPO-Li, at pH = 9 was UV irradiated to induce the photocopolymerization of AAPBA with the methacrylate groups of HPG-MA. As a result, the HPG-MA-PBA network containing HPG and PAAPBA subchains with MA groups as crosslinks was obtained. Since the concentration of AAPBA was rather low, some PAAPBA chains can be connected only to one MA group, forming grafted chains. In this network, in addition to irreversible crosslinks, one could also expect the formation of labile (reversible) crosslinks due to the interaction between PBA and the cis-diol groups of HPG [37]. Therefore, HPG-MA-PBA hydrogels contained two types of crosslinks: irreversible covalent crosslinks by MA groups and labile (reversible) crosslinks between PBA and HPG (Figure 6A).

Figure 6B shows the rheological properties of the prepared HPG-MA-PBA hydrogels. One can see that with increasing content of AAPBA from 0.2 to 2 wt%, the storage modulus increased by one order of magnitude, reaching ca. 30 kPa. This behavior suggested that at these conditions, the concentration of elastically active subchains in the network became higher.

Note that in HPG-MA hydrogels with only irreversible crosslinks, both G′ and G″ were almost frequency independent (Figure 5), but in most of the HPG-MA-PBA hydrogels, the loss modulus was highly frequency dependent, demonstrating marked maximum (G′′_max_) and minimum (Figure 6A). Such G′′(ω) dependencies are typical in polymer systems with labile crosslinks (e.g., temporary entanglements or weak bonds). For instance, such G′′(ω) dependencies are observed for poly(vinyl alcohol) (PVA) [49,50,51] and guar [18,19,52] gels crosslinked by borate. From the inverse value of the frequency ω* corresponding to G′′_max_, one can estimate the longest relaxation time (τ_rel_) that represents the time for a given macromolecule to disengage by reptation from a tube formed by neighboring chains [18]. Since the reptation in PVA/borate and guar/borate systems is hindered by diol/borate interactions, the longest relaxation time was considered as the reciprocal of the exchange rate for the formation of intermolecular crosslinks [18]. The ω* value depends on the number of diol/borate complexes, and it decreases when the diol or borate concentration increases [18].

A similar sticky reptation model can be valid for HPG-MA-PBA hydrogels if they contain free PAAPBA chains entrapped in the network or PAAPBA dangling chains linked to the network only at one end. In this case, reptation should be hindered because any monomer unit of PAAPBA can be reversibly linked to neighboring HPG chains. For 8 wt% HPG-MA-PBA hydrogel with 0.5 wt% PBA, the estimate of the longest relaxation time (τ_rel_) from the frequency ω* corresponding to G′′_max_ was 0.57 s. At higher PBA content, the clear maximum on G′′(ω) dependence disappeared. This may be explained, in particular, by a considerable slowing down of the relaxation process, which can be related to the formation of larger number of diol/borate links or to an increase in their lifetime as a result of the participation of several adjacent PBA units in the formation of crosslinks that makes the reversible crosslinks stronger and decreases their lability.

**Figure 7:**
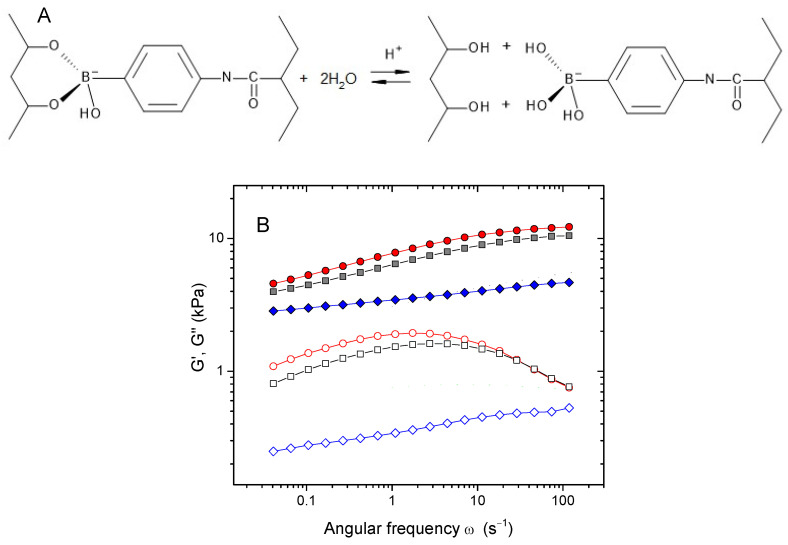
(**A**) Schematic representation of the disruption/formation of the links between cis-diol groups and PBA. (**B**) Frequency dependencies of storage G′ (filled symbols) and loss G″ (open symbols) moduli for 8 wt% HPG-MA-PBA hydrogels containing 0.56 mol% MA and 0.5 wt% AAPBA at different concentrations of added nitric acid: 0 (red circles), 0.87 mM (grey squares), and 42.1 mM (blue diamonds).

To reveal the impact of the labile crosslinks on the dynamic moduli, we destroyed the diol/borate crosslinks by decreasing the pH of the medium [53] (Figure 7A). Figure 7B illustrates the influence of added nitric acid on the viscoelastic behavior of the HPG-MA-PBA gels. It was seen that acid induced decreases in both dynamic moduli. This result clearly evidenced that the crosslinks produced by diol/borate interactions contributed significantly to the elastic modulus. Note that upon the addition of acid, the maximum of the G′′(ω) dependence disappeared, confirming that it was due to the presence of labile crosslinks.

Thus, HPG-MA-PBA hydrogels were prepared with two types of crosslinks: irreversible covalent crosslinks between MA groups and reversible crosslinks between PBA and HPG units.

### 2.4. pH-Controlled Swelling of the Hydrogels

**Figure 8:**
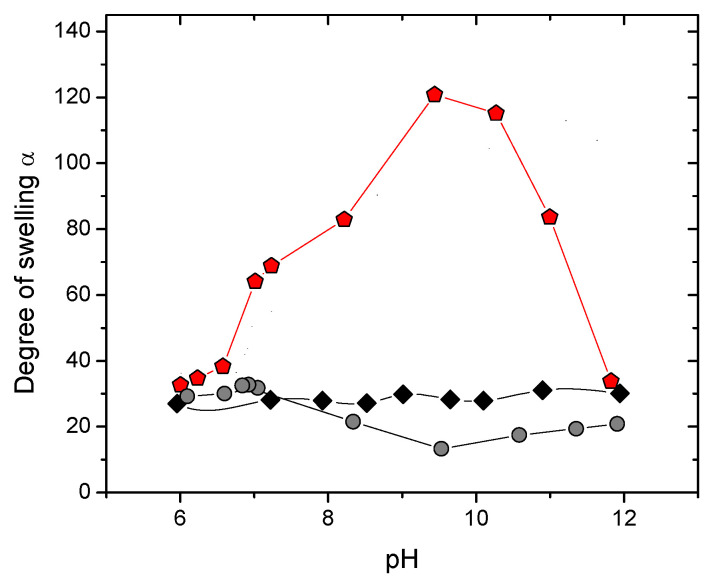
pH dependence of the degree of swelling (α) of the following hydrogels: HPG-MA hydrogel with 0.56 mol% MA in water (black diamonds), and HPG-MA-PBA hydrogel with 0.56 mol% MA and 2 wt% AAPBA in water (red pentagons) and in aqueous 0.9 wt% KCl (grey circles).

Additional labile crosslinks, on the one hand, strengthen the gel (the storage modulus increases); on the other hand, they impart pronounced responsive properties. Figure 8 illustrates the swelling behavior of the prepared hydrogels in water at different pH values. One can see that HPG-MA gels showed almost no dependence of the swelling degree on pH, as expected for a gel of a neutral polymer (just a small number of charges may arise from the initiator). In contrast, the behavior of HPG-MA-PBA gels was drastically different: they showed a prominent dependence of the swelling degree on pH. One can see that at pH 6, the swelling degree was close to that of the HPG-MA gel, since the PBA units were uncharged at this pH and did not contribute to swelling. When the pH exceeded ca. 6.8, the swelling degree dramatically increased, which can be explained by the progressive charging of PBA units leading to polyelectrolyte swelling arising from electrostatic repulsion between similarly charged monomer units and osmotic pressure exerted by counterions [1]. The maximum swelling degree of HPG-MA-PBA gels was ca. 120 (at pH 9.4), which exceeded the value for HPG-MA gels by a factor of 5. The maximum was located close to the pKa value of PBA, which is equal to 8.8 [54]. At higher pH, the swelling degree dropped, which may be attributed to the formation of additional crosslinks between AAPBA monomer units and HPG. In other words, two factors seemed to influence the swelling behavior of HPG-MA-PBA gels—charging of the AAPBA units, which favored swelling, and the formation of dynamic crosslinks between PBA and HPG, which can be regarded as additional effective attraction between HPG chains, favoring gel shrinking. When the pH was increased, first electrostatic effects prevailed and the gel swelled, but at a certain pH, when all AAPBA units were charged, the number of crosslinks increased and the gel shrank.

These conclusions were supported by the fact that the same HPG-MA-PBA gel in 0.9 wt% KCl solution did not show any increased swelling as compared to the HPG-MA gel (Figure 8). There was no maximum on the curve, and the swelling degree at pH values higher than 8 was lower than that for HPG-MA gel, which confirmed the formation of additional labile crosslinks and the absence of polyelectrolyte swelling in this case.

Thus, HPG-MA-PBA hydrogels exhibited pronounced pH-dependent swelling behavior.

### 2.5. pH-Controlled Release from the Hydrogels

The porous structure of hydrogels permits the loading of different substances into the gel matrix and then their release. Of particular interest is the possibility of creating gels that release disinfectants in response to specific triggers, like pH, that control the swelling behavior of the hydrogels related to pore size.

In the present study, we used the disinfectant CPC, which is effective, in particular, against SARS-CoV-2 [7]. CPC represents a cationic surfactant. Negatively charged HPG-MA-PBA hydrogels can effectively absorb oppositely charged surfactant ions because of ion-exchange reactions with polymer counterions. When the amount of the bound surfactant ions becomes close to that of the charged polymer units, the gel shrinks [55] since the surfactant ions aggregate in micelles, thereby decreasing the intranetwork osmotic pressure. However, the gel can further absorb the surfactant due to hydrophobic interactions. In this case, surfactant ions entered the gel together with their counterions, which induced an increase in osmotic pressure and reswelling of the gel [56]. To avoid gel collapse, we used an excess of CPC over the equimolar CPC/charged gel units ratio. Then, such gel with embedded antiseptic ions was cut into two pieces, one of which was immersed in water at pH 5.9 and the other immersed in water at pH 8.9. According to the swelling experiments (Figure 8), the gel should swell much more at pH 8.9 than at pH 5.9. As was discussed above, higher swelling is due to gel charging (pKa of PBA = 8.8 [54]).

**Figure 9:**
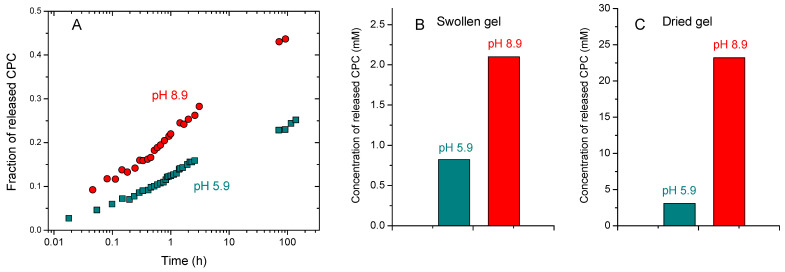
(**A**) Cetylpyridinium chloride release profile from 8 wt% HPG-MA-PBA hydrogels containing 0.56 mol% MA and 2 wt% AAPBA to 43 mL of water at pH 5.9 (green symbols) and 8.9 (red symbols). (**B**) Release of cetylpyridinium chloride from 8 wt% HPG-MA-PBA hydrogels containing 0.56 mol% MA and 2 wt% AAPBA swollen in water at pH 5.9 (green symbols) and 8.9 (red symbols) to 0.2 mL of 0.9 wt% NaCl solution in 2 s. (**C**) Release of cetylpyridinium chloride from dry coating prepared from 8 wt% HPG-MA-PBA hydrogels containing 0.56 mol% MA and 2 wt% AAPBA swollen in water at pH 5.9 (green symbols) and 8.9 (red symbols) to 0.2 mL of 0.9 wt% NaCl solution in 2 s.

Figure 9A shows the release profile of the disinfectant at different pH values (5.9 and 8.9). It is seen that the rate of release was much higher at alkaline pH, which may be due to higher gel swelling (Figure 8) favoring the diffusion of solute. The release profile followed a t^1/2^ time dependence, which is characteristic of Fickian diffusion [57]. One can see (Figure 9A) that the gel provided prolonged release of CPC over several days.

When considering the application of hydrogel coating for disinfection, it is important to study the release of disinfectant into small water droplets produced on the surface, for instance, by coughs or sneezes. To model this situation, we determined the amount of CPC released in 2 s to a very small volume (0.2 mL) of 0.9 wt% NaCl solution. The results are presented in Figure 9B. It is seen that, in this case, the release increased with increasing pH. The amount of released CPC at pH 8.9 was sufficient to completely inactivate SARS-CoV-2 (inhibition coefficient IC = 100%) in 5 s, as we previously demonstrated [7]. Figure 9C shows the results of the release of CPC from a dry coating prepared from the same HPG-MA-PBA gel to a very small volume (0.2 mL) of 0.9 wt% NaCl solution. In this case, the difference between the amount of CPC released at two different pH values became more pronounced. Most probably, this was due to the fact that the local swelling of the dried gel in contact with the added water droplet led to the release of CPC being much slower for the uncharged gel (at pH 5.9) than for the charged one (pH 8.9). As a result, in 2 s, much less CPC was released from the uncharged gel. At the same time, the concentration of the released CPC from the dried gel was much larger than that from the swollen gel (Figure 9B,C), which can be attributed to the higher concentration of CPC inside the gel when it was dried. Consequently, the amount of CPC released from the dried HPG-MA-PBA gels was more than sufficient to completely inactivate SARS-CoV-2 (inhibition coefficient IC = 100%) in 5 s [7].

Thus, the elaborated hydrogels demonstrated pH-dependent release of disinfectant, which can be used to produce antiseptic coatings with the desired activity.

Several polymer hydrogels have been proposed by now for the release of disinfectants, including, for instance, gels based on poly(4-vinylbenzyl chloride-co-acrylic acid) and poly(sodium 4-styrenesulfonate-co-glycidyl methacrylate) [58], gels based on copolymers of acrylamide and sodium 2-acrylamido-2-methylpropane sulfonate or sodium methacrylate [7], and microgels based on an interpenetrating network of poly(N-isopropylacrylamide) and polyacrylic acid [59]. The peculiarity of the present system is that (i) it is based on a biodegradable nontoxic polymer of natural origin and (ii) this polymer can be photocrosslinked in situ, which permits the creation of a long-lasting disinfecting coating on various (even very sophisticated) surfaces.

## 3. Materials and Methods

### 3.1. Materials

HPG (Jaguar^®^ HP-105) was kindly provided by Solvay (Brussels, Belgium) and used as received. It contained about 0.7 galactose side groups per mannose unit of the backbone and 0.91 moles of hydroxypropyl substituents per mole of monosaccharide units, as estimated by ^1^H NMR elsewhere [36].

Methacrylic anhydride (purity > 94%), AAPBA (purity > 98%), and CPC (purity > 98%) provided by Sigma-Aldrich (St. Louis, MO, USA) and TPO-Li (purity > 99%) obtained from CPS Polymers (Boulder, CO, USA) were used as received. The pH of samples was adjusted with potassium hydroxide (Acros, Geel, Belgium, 98%) or nitric acid (Sigma-Aldrich). Solutions were prepared with water purified and deionized on a Milli-Q system (Millipore Waters, Burlington, MA, USA). In the NMR experiments, deuterated water (AstraChem, Saint-Petersburg, Russia; isotopic purity > 99.9%) was used as a solvent.

### 3.2. Synthesis of Polymerizable HPG-MA Macromonomers

Methacrylated HPG-MA polymers were prepared by reacting 0.5 wt% aqueous solution of HPG with 0.65–2.46 mM methacrylic anhydride at pH 8 adjusted with 1 M NaOH. The reaction proceeded for 1 h at 4 °C. The polymers thus produced were precipitated and washed with 10-fold excess ethanol in order to purify them from unreacted methacrylic acid and methacrylic anhydride. Then, the synthesized HPG-MA polymers were dissolved in water, dialyzed, and lyophilized. This synthetic procedure was based on that proposed for the methacrylation of alginate, chitosan, and hyaluronic acid [41,42], but much smaller amounts of methacrylic anhydride and alkali were used.

### 3.3. Preparation of HPG-MA Hydrogels

To prepare the HPG-MA hydrogels, 15 mg of photo-initiator TPO-Li was added to 1.5 mL of 8 wt% aqueous solution of HPG-MA and mixed with a homogenizer for 15 min at a speed of 7000 rpm. Then, the solution was exposed to UV light (Irisk, Guangzho, China, power 36 W, wavelength 365 nm) for 3 min and a uniformly crosslinked gel was obtained.

### 3.4. Preparation of HPG-MA-PBA Hydrogels

To prepare the HPG-MA-PBA hydrogels, 15 mg of photo-initiator TPO-Li was added to 1.5 mL of 8 wt% aqueous solution of HPG-MA containing the desired concentration of monomer AAPBA at pH = 9 and mixed with a homogenizer for 15 min at a speed of 7000 rpm. Then, the mixture was illuminated with UV light (Irisk, Guangzho, China, power 36 W, wavelength 365 nm) for 3 min, yielding a hydrogel.

The effect of the decrease in pH on the rheological properties of the prepared HPG-MA-PBA hydrogels was studied by adding a small aliquot (0.26 and 12.6 µL) of 5 M HNO_3_ to 1.5 mL gels initially obtained at pH 9.

### 3.5. Gel Swelling

The degree of swelling of the gels was determined according to the formula [60]:

$$\alpha =\left(m_{sw}-m_{0}\right)∕m_{0}$$

where $m_{sw}$ is the mass of the swollen gel and $m_{0}$ is the mass of the dry gel.

### 3.6. Loading and Release of Disinfectant from the Hydrogels

In the first type of experiments, to load HPG-MA-PBA hydrogels with CPC, the hydrogel was immersed in 0.14 M aqueous solution of CPC for 24 h. Then, the gel was removed from CPC solution and cut into two pieces. One piece of gel was placed in 43 mL of water at pH 5.9 and the other was placed in the same volume of water at pH 8.9 (in both cases, the volume of water was about 200 times higher than that of the polymer gel). To study CPC release, small aliquots were taken from the solution surrounding the gel and diluted with distilled water to obtain the optical density (D) in the range from 0.1 to 1.

In the second type of experiments, to load HPG-MA-PBA hydrogels with CPC, 0.2 mL of a 0.244 M aqueous solution of CPC was poured onto the surface of the hydrogel. After one hour, 0.2 mL of 0.9 wt% aqueous NaCl solution was poured onto the surface of the gel and the solution was collected from the gel surface after 2 s, diluted with distilled water, and analyzed using UV spectroscopy.

In the third type of experiments, the loading of HPG-MA-PBA hydrogels with CPC was performed in the same way as in the second type of experiments. Then, the gel was dried at room temperature for 1 day. Afterwards, 0.2 mL of 0.9 wt% aqueous NaCl solution was poured onto the surface of the dried gel and the solution was collected from the gel surface after 2 s, diluted with distilled water, and analyzed using UV spectroscopy.

### 3.7. NMR Spectroscopy

^1^H NMR spectra were measured on a Bruker AV600 spectrometer (Billerica, MA, USA) in standard quartz ampoules (Norell) with a diameter of 5 mm using D_2_O as a solvent at 30 °C. The phase and baseline corrections of the spectra were performed using MestreNova software, version 14.2.1-27684, MestReLab Research S.L., Santiago de Compostela, Spain. The HOD signal of the solvent at 4.30 ppm served as a reference for determining chemical shifts.

### 3.8. Rheology

Rheological measurements were carried out using an Anton Paar Physica MCR 301 rotational rheometer (Graz, Austria) with a plate-plate geometry (diameter 25 mm, gap width 2 mm) and a casing preventing water evaporation. The temperature was maintained using Peltier elements at 20.00 + 0.05 °C. For the measurements, disc gel samples with a diameter of 25 mm and a height of 2 mm were prepared. The experiments were performed with gels just after synthesis. Before measurements, the samples were equilibrated in the measuring cell for 10–20 min. In the oscillatory shear experiments, the frequency dependencies of the storage G′ (ω) and loss G″ (ω) moduli were measured in the external frequency ω range of 0.001–100 s^−1^. Frequency sweeps were performed in the linear viscoelasticity mode with a strain amplitude of 1–5%. The experiments were performed as described in detail elsewhere [61].

### 3.9. Viscometry

A capillary viscometer was used to determine the reduced $\left(\eta_{red}\right)$ and intrinsic viscosity (η) of dilute polymer solutions. The pure solvent flow time $\left(t_{0}\right)$ was 81.30 ± 0.15 s. The flow time of each solution was measured 3 times. The reduced viscosity $\left(\eta_{red}\right)$ was calculated for each concentration of hydroxypropyl guar (C) as:

$$\eta_{red}=\frac{\frac{t_{i}}{t_{0}}-1}{C}$$

where $t_{i}$ is the flow time of polymer solution. To estimate the intrinsic viscosity ([η]), the reduced viscosity plotted as a function of the concentration of polymer (C) was extrapolated to infinite dilution.

### 3.10. UV Spectroscopy

UV spectroscopy studies were performed with U-2900 spectrophotometer (Hitachi, Tokyo, Japan). The CPC concentration (C_CPC_) was determined from the optical density (D) of the absorbance peak of the pyridinium ring at 259 nm as:

$$C_{CPC}=D∕\varepsilon l,$$

where ε is the extinction coefficient equal to 4070 L/mol·cm [7,55] and l is the length of the light path.

## 4. Conclusions

New double crosslinked hydrogels, HPG-MA-PBA, were prepared based on a biodegradable polysaccharide, HPG. They contain two types of crosslinks differing in strength and reversibility: strong irreversible crosslinks between MA groups and weak reversible crosslinks between PBA and HPG units. It was shown that additional labile crosslinks not only strengthen the gel by increasing its elastic modulus, but also impart pH responsiveness, which can be exploited to control the elasticity, the pore size, the swelling of the gels, as well as the uptake and release of some substances, for instance, disinfectants.

Since HPG-MA-PBA hydrogels are synthesized by photocrosslinking, they can be used to produce coverage of various surfaces in situ. Such coverage with controllable mechanical properties may serve as a smart matrix for prolonged release of disinfectants to limit the spread of various infections, including COVID-19.

## Data Availability

Data are contained within the article.
